# Suite of 3D test objects for performance assessment of hybrid photoacoustic-ultrasound breast imaging systems

**DOI:** 10.1117/1.JBO.27.7.074709

**Published:** 2021-12-09

**Authors:** Maura Dantuma, Saskia C. Kruitwagen, Marlies J. Weggemans, Tim J. P. M. Op’t Root, Srirang Manohar

**Affiliations:** a University of Twente, Multi-Modality Medical Imaging, Technical Medical Centre, Enschede, The Netherlands; b Medisch Spectrum Twente Hospital, Enschede, The Netherlands; c PA Imaging R&D B.V., Enschede, The Netherlands

**Keywords:** test objects, phantoms, image quality assessment, photoacoustic imaging, ultrasound imaging, breast imaging

## Abstract

**Significance:**

During the development and early testing phases of new photoacoustic (PA) breast imaging systems, several choices need to be made in aspects of system design and measurement sequences. Decision-making can be complex for state-of-the-art systems such as 3D hybrid photoacoustic-ultrasound (PA-US) breast imagers intended for multispectral quantitative imaging. These systems have a large set of design choices and system settings that affect imaging performance in different ways and often require trade-offs. Decisions have to be made carefully as they can strongly influence the imaging performance.

**Aim:**

A systematic approach to assess the influence of various choices on the imaging performance in carefully controlled laboratory situations is crucial before starting with human studies. Test objects and phantoms are used for first imaging studies, but most reported structures have a 2D geometry and are not suitable to assess all the image quality characteristics (IQCs) of 3D hybrid PA-US systems.

**Approach:**

Our work introduces a suite of five test objects designed for hybrid PA-US systems with a 3D detection aperture. We present the test object designs and production protocols and explain how they can be used to study various performance measures. To demonstrate the utility of the developed objects, measurements are made with an existing tomographic PA system.

**Results:**

Two test objects were developed for measurements of the US detectors’ impulse responses and light distribution on the breast surface. Three others were developed to assess image quality and quantitative accuracy of the PA and US modes. Three of the five objects were imaged to demonstrate their use.

**Conclusions:**

The developed test objects allow one to study influences of various choices in design and system settings. With this, IQCs can be assessed as a function of measurement sequence settings for the PA and US modes in a controlled way. Systematic studies and measurements using these objects will help to optimize various system settings and measurement protocols in laboratory situations before embarking on human studies.

## 1 Introduction

Photoacoustic (PA) tomography is an emerging non-invasive imaging technique that is making its entry into the clinics for applications in breast imaging.^1^^,^^2^ The technique enables the visualization of blood vessels deep in the breast with high resolutions using the intrinsic and wavelength-specific optical absorption contrast of hemoglobin in the blood.^3^^,^^4^ Depending on the configuration of the ultrasound (US) detection array, images with two or three dimensions can be generated. The pixel intensities in the resulting image depend, among others, on the concentration and the optical absorption coefficient ($\mu_{a}$) of hemoglobin in the blood.^5^ Quantitative photoacoustic (QPA) imaging^6^ seeks to map estimates of chromophore concentrations in tissue, which could be intrinsic, such as hemoglobin or lipids, or exogenous, such as contrast agents. This is achieved using multiple excitation wavelengths for PA imaging since chromophores often have wavelength-dependent optical absorption. Fluence compensation approaches, which can be algorithmic^7^^,^^8^ or experimental,^9^^,^^10^ are applied to achieve improved estimations of the concentrations. An estimation of the blood oxygen saturation (${\mathrm{SO}}_{2}$) is often the goal of QPA as it can provide insights into the breast and tumor physiology.

Over the last years, hybrid photoacoustic-ultrasound (PA-US) systems were being developed to provide structural and anatomic context from the US mode to improve interpretation of the PA images.^11^^–^^13^ Although most PA-US systems only provide US images based on reflection imaging, tomographic setups also allow one to additionally measure the spatial distribution of the speed of sound (SOS) inside the breast from transmission-mode US measurements.^14^ In addition to the diagnostic value of these SOS distributions in breast cancer imaging and breast density characterization,^15^^,^^16^ they can additionally be used as an input to the PA reconstructions to improve the image quality by accounting for the SOS differences in the breast.^14^^,^^17^

Developments of technology for hybrid PA-US breast imaging systems have been proceeding in all aspects of instrument design, hardware, and image reconstruction algorithms.^4^^,^^18^^,^^19^ It is interesting to note that there is no consensus in system design and measurement protocols used for PA-US breast imaging. At least four system geometries determined by the US recording aperture exist, namely: linear, planar, curved/circular, and hemispherical.^4^^,^^20^ At the same time, it is known that the system geometry has important implications for the performance of the imager.^19^ For example, the quantitation of ${\mathrm{SO}}_{2}$, a challenging problem, is expected to be more accurate as we collect more solid-angle projections around the breast. Another example is the illumination strategy and the choice of excitation wavelengths, which have implications on the PA imaging depth.^21^ Yet another example may be the choice of time sampling in the form of the number of signal averages.^22^ Oversampling will require longer measurement times and may not bring substantially improved resolutions or signal-to-noise ratios. It is, therefore, important to analyze the implications on the imaging performance of design choices as well as measurement setting choices.

A clinical study with a new system has to be preceded by a period of comprehensive system testing to improve the system design, to assess and optimize the imaging performance, to evaluate if the system complies with safety regulations, and to train the users.^23^^,^^24^ To streamline this process, three phases are usually followed. (i) In the first phase, verification measurements on the system modules will inform if the system functions as it was designed. In practice, this means that test measurements need to be performed to check if the various modules (such as the US detectors, laser, and data-acquisition electronics) individually perform to the technical specifications according to which they were designed. Knowledge about the performance of the individual US detection elements, as the sensitivity and frequency response, can help in estimating the system’s imaging performance. In addition, implementation of the detector characteristics into the image reconstruction algorithms will also improve the image quality.^25^^,^^26^ Knowledge about the fluence on the skin is required for laser safety evaluations and can be used in developing accurate optical inversion (OI) models that can be used for fluence compensation of reconstructed PA pressures for estimating ${\mathrm{SO}}_{2}$ with QPA.^27^ (ii) The second phase usually consists of a set of measurements where the image quality characteristics (IQCs) of the system are assessed and benchmarked under methodical changes of the measurement sequence and parameters in the reconstruction algorithms.^28^^–^^30^ Measurement settings resulting in optimized IQCs with a minimum required measurement time to minimize motion artefacts and burden to the patient are hereby the target. (iii) In the third phase, measurements on more complex objects, such as breast mimicking phantoms or healthy breasts, are performed to test the system to a higher extent and to train the users.^31^^,^^32^

Especially, 3D tomographic multispectral PA-US systems come with many programmable measurement settings, and phase (ii) can be challenging. Next to the number of projections and the number of PA averages, a decision may have to be made regarding the choice of emitter–receiver pairs for US computed tomography. For QPA, a selection on the excitation wavelengths has to be made and one should decide on the number of measurements that are performed per wavelength. Unravelling the optimal measurement sequence for these kinds of systems from measurements on healthy or affected breasts is non-trivial due to the unknown ground truths. All of this points to the requirement for a set of inanimate objects that can be used in a laboratory setting and that provide the ground truth for the various IQCs sought after by the imaging system. By imaging these objects under various settings and combinations of settings, optima can be found to achieve the sought-after optimal IQCs. These test objects and phantoms will necessarily have to be well-characterized for their geometrical, optical, and acoustic properties.

### 1.1 Existing Test Objects

Several test objects with prescribed physical properties for the characterization of PA imaging systems have been reported in the literature over the years. To measure the PA spatial resolution of systems, subresolution targets as absorbing spheres, epoxy dots, black hairs, or wires^31^^,^^33^^–^^35^ are often used. Wires or channels filled with blood mimicking liquids located at different depths in a tissue-mimicking material have been reported multiple times to measure the imaging depth of PA systems with a linear or planar detector array.^28^^,^^35^^–^^38^ As an approach to measure the imaging depth of a system with a 3D imaging aperture, a transparent disk printed with red dots was placed at different heights in an acoustically absorbing medium.^32^^,^^39^ To assess the ability of a system to resolve an object from the background, targets with different optical absorption coefficients have been embedded in a material with tissue relevant properties.^36^^,^^40^

As more systems are nowadays equipped with two or more illumination wavelengths, the demand for test objects for QPA has increased. Recent works on blood vessel phantoms have been reported,^41^^–^^43^ where channels in tissue mimicking materials were flushed with blood. Connecting these vessels to a pump and a flow spectrometer allowed to monitor the blood ${\mathrm{SO}}_{2}$ in real time.^42^ The ${\mathrm{SO}}_{2}$ could be regulated chemically by the addition of sodium hydrosulfite^42^ or with a membrane oxygenator.^43^

As diagnostic US imaging has a 60-year long history,^44^ a wide variety of phantoms have been reported for this imaging modality as well.^45^ Most phantoms were developed for testing linear arrays but with the returning interest in the US tomography^46^ for breast imaging, more 3D phantoms are being developed. A first rather complex breast mimicking phantom was already reported in 1982 by Madsen et al. The phantom consisted of different layers mimicking fat and fibro glandular tissue, and the phantom contained multiple tumor inserts.^47^ More recently, Duric et al.^30^ used a systematic approach to test the capabilities of their in-house built US tomography prototype with measurements on progressively more complex agar-based objects. The first object contained nylon wires as microcalcifications and in the second inclusions were filled with a water/alcohol mixture to represent cysts. Jose et al.^48^ also developed similar cylindrical agar-based objects, containing inserts to test US tomography in a table-top hybrid tomographic US-PA imaging prototype.

Although most studies, both in PA and US imaging, only demonstrate the characterization of a few IQCs, Vogt et al.^36^ were the first to publish a set of test objects to assess a range of important IQCs of hand-held PA systems. They presented four test objects for assessing the imaging resolution, uniformity, spatial accuracy, sensitivity, penetration depth, and PA-US co-registration accuracy. Later, the same group also reported on a set of objects for the performance evaluation of multispectral PA hand-held systems.^28^ Although these objects have great value for systems with linear arrays, they are not all suitable for 3D tomographic breast imaging systems such as reported in Refs. 31, 32, 49, and 50 since these systems specifically require objects with 3D geometries.

In the previous works,^41^^,^^51^ we introduced advanced breast-mimicking phantoms for testing PA and QPA algorithms, which will be valuable in phase (iii) of the system testing process. In this work, we introduce test objects to use in phases (i) and (ii). With this, we introduce a suite of test objects for the performance assessment of state-of-the-art 3D multispectral PA-US tomography systems that use the US modality in transmission mode for estimating the SOS in the breast. We present a list of functional requirements based on which the test objects were designed. To minimize the required number of measurements in the optimization process, it was attempted to combine several features in each test object. This resulted in two objects for module specification verification [phase (i)]. With these, the system’s illumination and detection characteristics can be measured and this knowledge can be implemented in the PA and US reconstruction and QPA algorithms. Three other objects were designed to assess the PA spatial resolution and imaging depth, the SOS spatial resolution and accuracy, and the ${\mathrm{SO}}_{2}$ estimation accuracy in phase (ii). The design and production protocols of the five objects are described, and the utility of three of the objects is demonstrated. In the absence of state-of-the-art 3D hybrid PA-US CT systems that are still under development,^52^ the available PA CT imager PAM 2^31^ was chosen to demonstrate the applicability of the test objects. This system has two excitation wavelengths for full breast PA imaging but is not equipped with the US modality. This manuscript, therefore, only presents the utility of the objects for the PA mode, but image acquisitions and reconstructions have been performed in such a way as to demonstrate the influence of excitation wavelength and the SOS used in the PA reconstruction on PA image quality.

## 2 Materials and Methods

### 2.1 Functional and Design Requirements of the Test Objects

In this section, the requirements for test objects to be used in phases (i) and (ii) are described. For phase (i), we focus on the verification of the light delivery and US detection characteristics. These are the two principle system hardware modules in PA imaging and have important implications on the imaging performance since they are responsible for the excitation and detection of the signals to enable PA imaging.^4^ Characteristics of these modules can additionally be used in PA reconstruction algorithms to model US sensing for improved acoustic inversion and to model light incidence on the breast for the OI. For assessment of the PA image quality, as part of phase (ii), we focus on the spatial resolution and imaging depth, as these are performance characteristics that report on the detail and extent to which the breast volume can be imaged. Errors in sound speed distributions used in the acoustic inversion model can lead to compromised PA imaging resolutions and contrasts^14^^,^^17^ and should, therefore, be minimized. Blurred SOS distributions, as a result of limited imaging resolutions, affect the quantitative accuracy of the SOS estimation. Therefore, test objects that allow one to assess both the quantitative accuracy (SOS accuracy) and the spatial resolution (SOS resolution) should be developed. Finally, the accuracy of the ${\mathrm{SO}}_{2}$ estimations made with QPA should be made assessable. This allows one to optimize the QPA codes until errors in the estimations are minimized.

The requirements for test objects for use in phases (i) and (ii) are listed below. An overview of all the IQC and test object requirements is presented in Table 1. In addition to the specific requirements, general considerations are also kept in mind. Prime among these is that the materials used should possess stable optical and acoustic properties over time and are compatible with water used as the coupling medium during measurements.

**Table 1:** Requirements for the test objects to assess the most important IQCs of the PA images, the SOS maps that are obtained with the US modality, and the blood oxygen saturation (${\mathrm{SO}}_{2}$) maps acquired with QPA.

Modality	IQC	Phantom requirements
PA	Spatial resolution	1. Subresolution PA targets
		2. PA targets located at or translatable to different positions in the imaging volume
		3. Optically and acoustically semitransparent fixation method
	Imaging depth	1. Targets with $\mu_{a}=\mu_{a,\text{blood}}$
		2. Targets located at or translatable to different depths in the imaging volume
		3. A tissue-mimicking embedding material
		4. Targets should not block the light for possible other targets
US	SOS spatial resolution	1. Subresolution SOS targets or step-change SOS contrast at different positions in the imaging volume
		2. Low acoustic attenuation to remain with detectable pressure amplitudes after transmission
	SOS accuracy	1. Material(s) with well-characterized sound speed(s)
		2. No pointy shapes, to minimize diffraction artifacts
QPA	${\mathrm{SO}}_{2}$ accuracy	1. Blood or blood-mimicking material with known ${\mathrm{SO}}_{2}$ value as targets
		2. Targets located at or translatable to different depths in the imaging volume
		3. Tissue realistic embedding material

#### 2.1.1 Phase (i): module specification verification

1. *US detector behavior*. To quantify the response of all US detection elements in the system, a broadband acoustic source is required. Here, one could think of PA subresolution targets or alternatively larger targets with a subresolution optical thickness irradiated with nanosecond light pulses to comply with thermal and stress confinement.^53^ For measurements of the relative sensitivity of all elements, the target must be located equidistant to all elements, and such that the PA waves have normal incidence to the detectors. The object should also be illuminated as homogeneously as possible to expose all detecting elements to the same absolute PA pressure.
2. *Light fluence distribution on the breast*. In PA systems with 3D detection apertures, breasts are often supported by a stabilizing cup.^31^^,^^32^^,^^49^^,^^54^ When a cup size is selected that fits the breast, the organ takes the shape of the cup, which then accurately defines the breast contour. A test object should, therefore, be developed that allows one to investigate the light distribution on the contours of these breast supporting cups.

#### 2.1.2 Phase (ii): image quality characteristic assessment

1. *PA spatial resolution*. The spatial resolution can be calculated by measuring the full-width half-maximum (FWHM) of a Gaussian fitted to a point spread function (PSF) obtained from measurements on subresolution PA sources. The imager’s PSF is often spatially variant and anisotropic as a result of the geometry of the detector array and the reception apertures of the individual detectors. To characterize the PSF throughout the imaging volume and for all imaging planes, subresolution PA sources should be scanned through the imaging space, or alternatively multiple of these sources should be placed at several locations in the imaging volume. The used fixation method should minimally disturb the generated PA waves and to ensure that the sources generate detectable PA amplitudes at every location in the imaging volume, the background medium should be acoustically and optically semitransparent.
2. *PA imaging depth*. The PA imaging depth strongly depends on the acoustic and optical attenuation properties of the breast tissue. To measure the imaging depth of a system, the object should consist of a material with breast tissue realistic optical and acoustic properties. Second, targets with an optical absorption coefficient ($\mu_{a}$) equal to the $\mu_{a}$ of blood should be embedded at different depths within the object. Here the targets should ideally not block the light travelling to the deeper-lying targets.
3. *SOS spatial resolution*. To assess the spatial resolution of the SOS maps resulting from the US transmission measurements, we again need subresolution targets of which the FWHM can be measured in the reconstruction. Instead of optically absorbing targets that are required for measuring the PA spatial resolution, the SOS spatial resolution has to be assessed with targets that have an SOS contrast with the surrounding medium. Instead of looking at the reconstructed PSF of subresolution targets, it is also possible to measure the spatial resolution from discrete jumps in SOS (sharp material interfaces). In this case, the spatial resolution can be estimated from the step in sound speed at the location of the interface in the reconstruction. Finally, the acoustic attenuation coefficient of the used materials should be sufficiently low such that a detectable amplitude remains after transmission through the object.
4. *SOS accuracy*. To validate the accuracy of the SOS estimations, the object must consist of materials with well-known sound speeds. To minimize diffraction artefacts from occurring, which induce errors in the SOS estimations by altering the path length a wave travels, the object should not contain any pointed shapes.
5. ${\mathrm{SO}}_{2}$ *estimation accuracy*. To measure and optimize the accuracy of the system in recovering ${\mathrm{SO}}_{2}$ estimations with QPA, the test objects should contain blood or blood-mimicking materials with well-known ${\mathrm{SO}}_{2}$ values. To validate the OI models that are part of the QPA algorithms, the ${\mathrm{SO}}_{2}$ should ideally be controllable and the targets should be located at different locations inside the imaging volume. The supporting material should have known tissue realistic optical and acoustic properties.

### 2.2 Measurements on the Test Objects

#### 2.2.1 Optical and acoustic material properties

The relevant optical and acoustic properties of the materials that the test objects are built from were characterized. Acoustic sound speeds and attenuation were measured with US transmission measurements following the method described in Ref. 51. The optical absorption of non-scattering media was calculated from transmission measurements in a spectrophotometer following the Lambert–Beer law. Optical properties of scattering media were calculated from transmission and reflection measurements in the same spectrophotometer with the inverse adding doubling method.^55^ The most relevant acoustic and optical material properties are included in the test object descriptions in the results section. Additional material properties can be found in Fig. S4 in the Supplementary Material.

#### 2.2.2 Photoacoustic imaging

The test objects were imaged with the Twente PAM 2^31^ to demonstrate their PA appearance and show their potential use. The PAM 2 system is a tomographic system that is equipped with a dual-head laser, emitting at 755 and 1064 nm. A bottom beam illuminates the breast from the nipple side and nine fiber bundles illuminate the breast from the sides. The system contains a 1-MHz-centered hemispherical detection aperture that consists of 12 arc-shaped arrays. The arrays are radially positioned and rotate around their central coordinate during a measurement to obtain multiple projections. PA images were reconstructed from the recorded time traces with an iterative back-projection algorithm. A detailed description of the system and the reconstruction algorithms can be found in Refs. 31 and 54. The well-known test object shapes allowed one to employ a two-layer SOS model in the reconstruction, where one SOS was assigned to the coupling water and one to the test object volume. The SOS assigned to the water was determined by the water temperature that was measured for each measurement. The SOS assigned to the test object volume varied per test object and reconstruction and is specified with the images in the next section.

## 3 Results

### 3.1 Descriptions and Implementation of the Test Objects

**Fig. 1:**
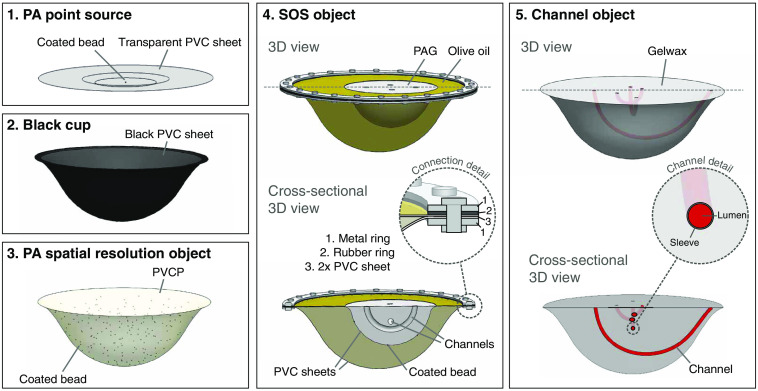
Illustrations in 3D of the test object suite showing their most important features. PA, photoacoustics; PVC, polyvinylchloride; PVCP, polyvinylchloride plastisol; and PAG, polyacrylamide gel.

**Fig. 2:**
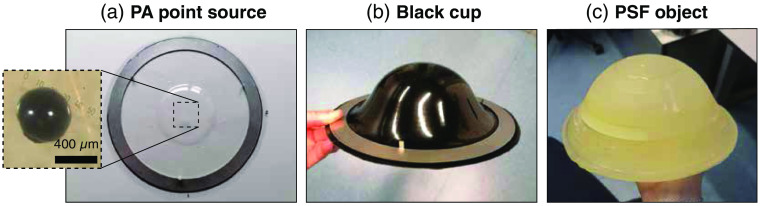
Photographs of test objects. (a) The single PA source attached to a PVC sheet. A bright-field microscopy image of the bead is shown in the inset. (b) One of the eight produced black cups (size 6). A metal ring is attached to the cup to mount it in the imaging aperture. (c) The PA spatial resolution object, showing the PVCP layers.

**Fig. 3:**
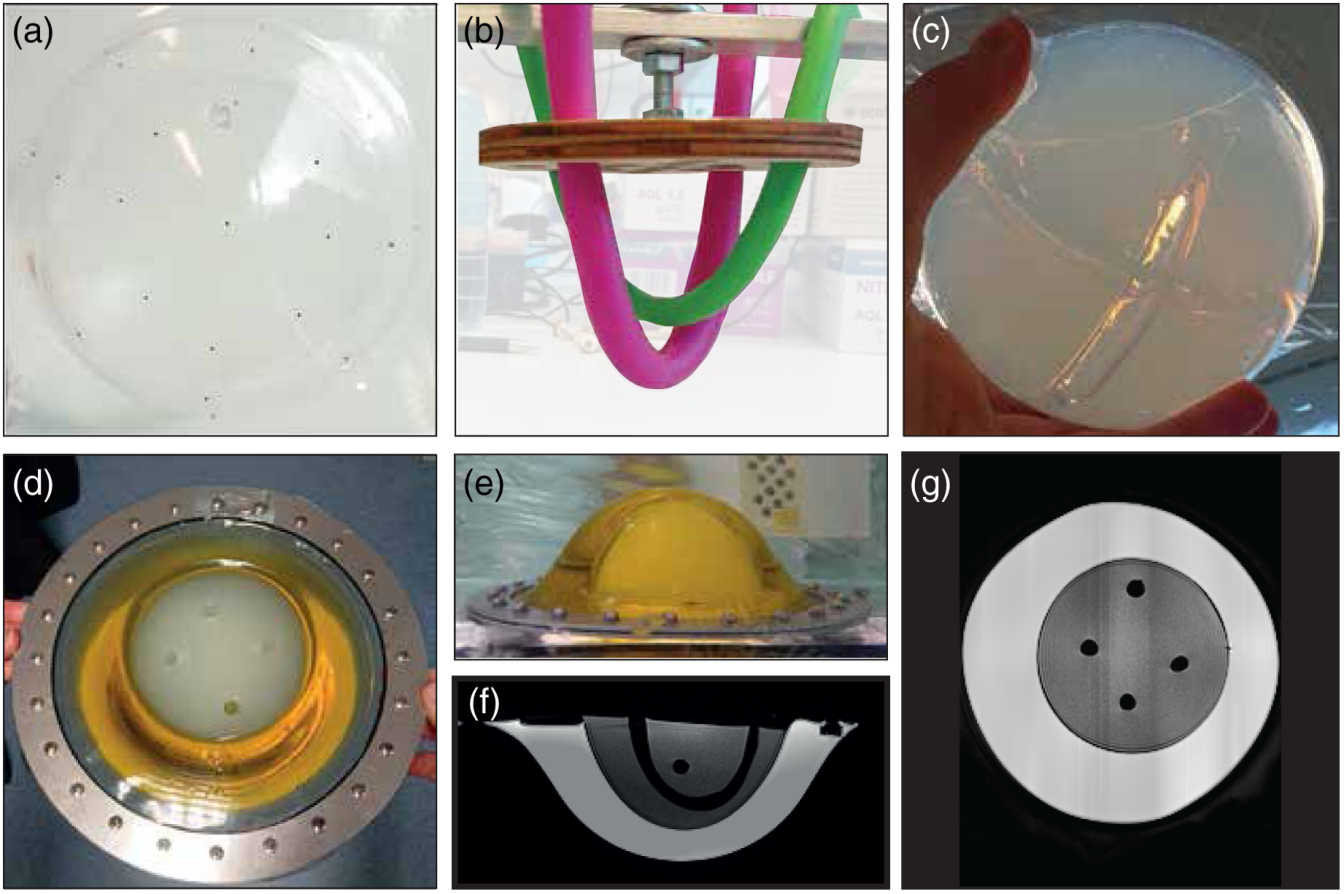
Pictures taken during and after the production of the SOS object. (a) Subresolution beads glued to the ellipsoid cup. (b) Modeling balloons filled with water as placeholders for the channels in the PAG. (c) The polyacryl amide gel volume after draining and removing the balloons. (d), (e) Top view and side view of the assembled SOS object, showing the olive oil and PAG compartments. The channel openings in the PAG are also visible. (f), (g) Cross sections of an MRI scan made of the object, showing the object’s build-up.

**Fig. 4:**
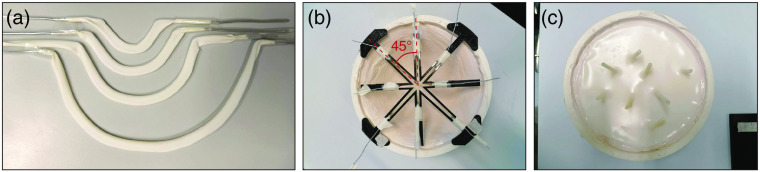
Pictures taken during and after production of the channel object. (a) The four bent metal wires covered in the US sleeves. (b) The channel shapes positioned in the cup eight shaped mould using a holder. (c) Top view of the channel phantom after pouring in the gel wax and removal of the holder and the metal wires. The channel sleeves protrude from the object and can optionally be connected to a flow circuit.

Considering all the requirements, five test objects were designed and developed. We tried to combine as many features into each object, to reduce the number of required measurements in the optimization process. Two test objects for system module testing, a single PA source and black cups were developed for phase (i). A PA spatial resolution object, SOS object, and channel object were developed to assess the IQCs in phase (ii). The designs of the test objects are depicted in Fig. 1 and will be described one by one in this section together with their production protocols. Possible implementations for each of the objects are also described and an overview of all their individual applications in system testing and/or image quality optimization is summarized in Table 2. Photographs of the test objects are presented in Figs. 2–4.

**Table 2:** Test object descriptions and their utilities in phase (i) module specification verification and phase (ii) IQC assessment. For phase (ii), the IQCs to assess are given together with the variables that are expected to influence them. The measurement sets that are presented in this paper are marked in bold. US, ultrasound; PA, photoacoustics; OI, optical inversion; SOS, speed of sound; QPA, quantitative photoacoustics; ${\mathrm{SO}}_{2}$, blood oxygen saturation; and ROC, radius of curvature.

No.	Object	Description	Phase	Function
1	PA point source	Subresolution absorber on transparent disc; absorber equidistant to all US detection elements	(i)	1. First PA measurement
				2. Measure detector characteristics
				3. Update PA inversion codes
				4. Monitoring detector performance
**2**	**Black cup**	Black breast supporting cup	**(i)**	**1. Visualize light distribution on skin**
				2. Update OI models
**3**	**PA spatial resolution object**	Subresolution absorbers distributed in imaging volume; supported by a semitransparent material	**(ii)**	**Assessment of PA spatial resolution**
				– **Spatial coordinate**
				– No. of PA detector positions
				– No. of PA averages
				– PA reconstruction parameters
4	SOS object	Object with volumes of different but known sound speeds; embedded with subresolution PA targets	(i)	1. First US measurement
				2. Using SOS map for PA inversion
				3. PA and US co-registration
			(ii)	Assessment of SOS spatial resolution and SOS accuracy
				– No. of US emitters
				– No. of US detectors
				– No. of detector positions
				– No. of US averages
				– US reconstruction parameters
**5**	**Channel object**	Object of tissue mimicking material with four channels, each with a different ROC; placed at 45 deg to each other in xy plane; channel-surface distance increases along channel path; the smaller channel starts at a depth where the larger one ends	**(ii)**	**Assessment of the PA imaging depth**
				– No. of PA detection positions
				– No. of PA averages
				**– Wavelength**
				– PA reconstruction parameters
				Assessment of the ${\mathrm{SO}}_{2}$ accuracy
				– Depth
				– No. of PA wavelengths
				– Wavelengths used
				– Parameters in QPA code

#### 3.1.1 PA point source

A single subresolution PA point source was developed, which can function as an object for early system testing in phase (i) to validate the data acquisition and PA reconstruction algorithms. Its second use lies in the measurement of the detection bandwidth and relative sensitivity of all the US detection elements. As a third use, the object can also be used in monitoring the detector performance over time. The basis of this test object is a stainless steel microsphere with a 310- to 360-μm diameter (Cospheric LLC, United States). A microscope image of these beads is included in Fig. S1 in the Supplementary Material. The beads were dyed by dipping it in India ink (Royal Talens, The Netherlands) and letting it dry at room temperature. The inset in Fig. 2(a) shows a microscope image of a coated bead.

A thin (180  μm) circular PVC sheet (Krumbeck Kunststoffverarbeitung GmbH, Stadtlohn, Germany) was vacuum formed over an 8.7-mm-thick silicone disk. The bead was then glued to the center of the PVC sheet using a droplet of superglue (Loctite 406, United States). This enabled positioning of the microsphere at the central coordinate of the hemispherical detection aperture of the PAM 2 system, equidistant to all detectors while using the standard cup mounting system. The PVC sheet is known to minimally disturb the PA measurement due to its high optical and acoustic transparency.^54^

#### 3.1.2 Black cups

As a second test object, we developed breast supporting cups in eight different sizes from black PVC. The starting material was a 1±0.1-mm-thick black PVC-U sheet (SIMONA AG, Germany), which was vacuum-formed using a vacuum shaping machine (Formech 300, Formech International Limited, UK). The same moulds that are normally used to produce the transparent breast supporting cups^54^ for the PAM 2 system were used. The reconstructed pressures from PA measurements on these objects will be a representation of the illumination profile on the breast surface. With this knowledge, the OI models for QPA can be made cup-specific.

#### 3.1.3 PA spatial resolution object

The PA spatial resolution is an IQC that can potentially be optimized by changes in the measurement sequence or improvements made in reconstruction algorithms. A test object containing 277 subresolution India ink coated stainless steel microspheres spread out over the imaging volume was developed to measure the spatial resolution in all three dimensions and through the entire imaging volume. The microspheres were held in a polyvinyl plastisol (PVCP) matrix with a 1403±3  m/s sound speed at 1 MHz. No scatterers or absorbers were added to the PVCP to keep it optically and acoustically semitransparent. A Nylon (PA2200) mould in the shape of the largest breast supporting cup was 3D printed with the Formiga P101 printer (EOS, Germany) (see Fig. S2 in the Supplementary Material). Nylon was chosen as it is known to withstand the high temperature of the liquid PVCP (±180°C). The large volume of this mould (1.2 l) and the fact that large volumes of PVCP tend to get overheated and burn during the polymerization process led us to build this phantom layer-by-layer. For each layer, a volume of PVCP (LureFlex firm, LureFactors, UK) was measured and 1 v/v% heat stabilizer (M-F Manufacturing, Texas, USA) was added to make it better resistant to heat. The heat stabilizer was mixed through the PVCP for 5 min with a magnetic stirrer. To assure an equal layer thickness, the PVCP volumes ranged from 50 to 400 ml, where the smaller volumes were used for the first layers in the bulge of the cup mould. The opaque PVCP mixtures were degassed for 5 min and heated in the microwave at 270 W in steps of 1 min until the mixture transformed into a transparent viscous liquid. The mixture was slightly swirled but not stirred to prevent air bubbles from forming. Subsequently, the PVCP was poured into the mould, leaving the not fully polymerized PVCP in the beaker. Directly after pouring, several stainless steel beads were distributed on the surface [see Fig. S2(b) in the Supplementary Material]. This process was repeated 11 times to fill the mould to the top. The 11th layer acts as a shutoff layer to protect the beads in the 10th layer. The object was placed in a size 8 breast supporting cup to install it in the imager.

#### 3.1.4 Speed of sound object

A test object containing materials with different sound speeds was developed to test the ability of the US transmission modality in calculating SOS maps. The inclusion of PA absorbers in this test object allows one to investigate improvements in the PA spatial resolution when the object’s sound speed, known *a priori* or measured with US transmission measurements, is used in the PA reconstruction.^14^^,^^48^^,^^56^

The object was once again given the shape of the largest breast supporting cup and includes sections that have different sound speeds. The material interfaces were given a gradually curved shape to minimize diffraction artifacts. The test object was built from two thin transparent (180  μm) vacuum-formed PVC cups (Krumbeck Kunststoffverarbeitung GmbH, Stadtlohn, Germany) that were put together. The outer cup is a size 8 breast supporting cup, and the therein placed cup is a smaller 3D ellipsoid and is positioned off-center. The same India ink coated microspheres as used in the other test objects were glued to the outside of the ellipsoid cup [Fig. 3(a)]. The volume between the two cups was filled with degassed traditional olive oil (Carbonell, Spain) with a 1459±3  m/s sound speed, forming the outer layer. Two bolted metal rings were used to clamp the two PVC sheets and a rubber ring together to avoid leakages of the oil. The hemispherical cup was then filled with polyacrylamide gel (PAG) with a 1512±3  m/s sound speed that was prepared by mixing 82.08 v/v% MiliQ, 17.5 v/v% acrylamide-bisacrylamide (VWR, USA, catalog number SERA10679.02), and 0.14 w/v% ammonium persulfate (Sigma Aldrich, USA, catalog number 248614).^57^ Two modeling balloons (Modelleerballon, Art. No. 10057434, Fun & Feest, The Netherlands) filled with water were used as placeholders for channels [Fig. 3(b)] and were positioned in the mixture. Finally, 0.28 v/v% tetramethylethylenediamine (Sigma Aldrich, USA, catalog number T22500) was added to start the polymerization process. After hardening of the PAG, the water-filled balloons were drained and removed [Fig. 3(c)]. The resulting wall-less channels with an 8-mm diameter can be filled with liquids with varying sound speeds. This allows one to not only investigate the SOS spatial resolution and accuracy but also the SOS contrast resolution and the effect of large impedance mismatches on the US reconstructions. Figures 3(d)–3(g) show pictures of the object and MRI cross sections of the object acquired with a Siemens Magnetom Aera 1.5T scanner.

#### 3.1.5 Channel object

A test object consisting of a breast tissue-mimicking material with four embedded channels was developed to investigate the PA imaging intensity and the QPA performance as a function of depth. All channels have another radius of curvature and are placed under 45 deg separation angles with respect to each other in the xy plane to minimize shadowing effects from the shallower channel(s) on the deeper-lying channel(s). The distance from the channels to the object’s surface gradually increases, and the deeper-lying channel starts at a depth where the more shallow one ends. This together with the rotational symmetry of the PA system enables the extraction of continuous information about the depth dependence of the IQC from 5 to 60 mm. Connection of the channels to a flow system equipped with a blood oxygenator allows flushing the object with blood and to regulate the blood oxygenation levels as in Refs. 41 to 43.

To give the channels the designed curvature, a plate with ridges in the designed channel shapes was 3D printed from PLA. Metal wires (1 mm) were bent along these ridges. US probe sleeves with a 5-mm diameter (Microtek Medical B.V, The Netherlands, catalogue number G88500) were spanned over the metal wires and will later form the walls of the channels and prevent diffusion of blood or other liquids into the test object [see Fig. 4(a)]. The same nylon breast mould introduced for the PA spatial resolution object was also used to shape this test object. The channel shapes were positioned in this mould using a holder [see Fig. 4(b)], and the mould was topped off with a gel wax mixture, which was prepared according to the methodology also used by^58^^–^^60^ using 93.5 w/w% native gel wax (Mindset, UK), 6 w/w% with paraffin wax (Sigma Aldrich, USA, catalog number 327204), 0.5 w/w% glass beads (≤106  μm, Sigma-Aldrich, USA), and 0.5 w/v% ${\mathrm{TiO}}_{2}$ (Sigma-Aldrich, USA, Catalog No. 248576). The glass beads and ${\mathrm{TiO}}_{2}$ were added for acoustic and optical scattering, respectively. After solidification of the gel wax, the metal wires were gently removed while leaving the sleeves in place [see Fig. 4(c)]. Finally, the entire object was removed from the mould and the object was placed in a size 8 breast supporting cup to install it in the imaging aperture. The sound speed and acoustic attenuation of the gel wax mixture were measured at 1 MHz and yielded 1445±1  m/s and 0.35±0.1  dB/cm, respectively. Optical absorption and scattering were also measured. The optical absorption yielded 0.50 and $0.81\;{\mathrm{cm}}^{-1}$ at 755 and 1064 nm, and reduced scattering coefficients of 14.76 and $9.78\;{\mathrm{cm}}^{-1}$ were found. See also Fig. S4 in the Supplementary Material for how the measured properties relate to properties that can be expected in a real breast.

### 3.2 Imaging Results

Imaging results from black cups, the PA spatial resolution object and the channel object are included and thoroughly described in this section as they possess the most interesting PA features. The PA images from measurements on the single PA source and the SOS phantom are presented in Figs. S1 and S3 in the Supplementary Material for demonstration purposes but are not further discussed.

#### 3.2.1 Black cups

**Fig. 5:**
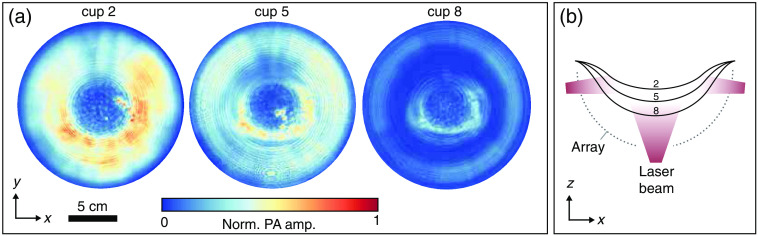
(a) MIPs along the z direction of the PA images from measurements on black cup 2, 5, and 8. The projections of the cups are normalized to the same value to illustrate absolute fluence differences between the cup sizes. (b) A schematic of the PAM2 illumination geometry showing the contours of black cups 2, 5, and 8.

Black cups with sizes 2, 5, and 8 were placed in the PAM 2 system and were imaged with 1064 nm. The cups were filled with water to assure acoustic coupling on both the top and underside of the cup. Images were reconstructed using a 1-SOS model (1496  m/s). Figure 5 shows the reconstructions of the measurements as maximum intensity projections (MIPs) along the z axis. The three projections are normalized to the same maximum value to illustrate absolute amplitude differences on the surface of the cups. With increasing cup size, lower amplitudes are reconstructed since the light is distributed over a larger surface area. For all cups, a low-intensity circle is observed in the center, which may be caused by a lack of detectors around the rotational axis of the PAM 2 system as this space is occupied by the bottom illumination beam. The side illumination beams create a ring-shaped profile on the cups, which can be observed on cup eight, whereas the bottom beam illuminates the cups in the central region. Smaller cups achieve a more homogeneous illumination profile since larger distances between the laser fibers and the cup surface allow the laser beams to diverge more. For the smaller cups, this means that the two illumination regions overlap (see cup 2), whereas they are separated for the larger cups (see cup 8). Homogeneous distributions over the entire cup surface will have the advantage that PA images of the breast volume are obtained with a relatively large field of view and with PA amplitudes that decrease with depth as can be expected from the tissue optical and acoustic properties. Inhomogeneous light distributions in contrary can result in PA images with limited field of views or with PA amplitudes that not only scale with the depth from the skin but also with the spatially varying intensities on the skin surface.

#### 3.2.2 PA spatial resolution object

The PA spatial resolution object was imaged with 1064 nm, and an image was reconstructed using a 2-SOS model where 1496  m/s was assigned to the water and 1413  m/s to the volume within the cup, matching to the SOS of the PVCP. To demonstrate the effect of errors in sound speeds assigned to the grid in the PA reconstruction on the PA resolution, a second reconstruction was made where 1496  m/s was assigned to the entire imaging volume. The PA spatial resolution was calculated for both reconstructions for 84 PA targets. These 84 targets were the targets with no other targets in their close surroundings to allow accurate fitting of the Gaussians.

**Fig. 6:**
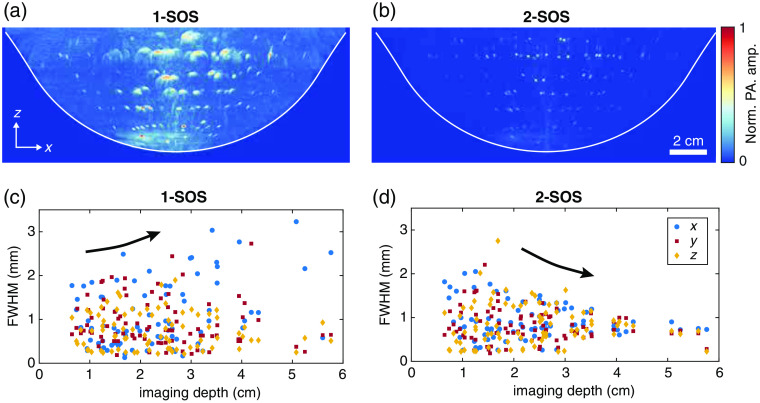
(a), (b) MIPs along the y axis for 1-SOS and 2-SOS reconstructions. Both MIPs are normalized to their own maximum pixel value. (c), (d) The FWHM from the Gaussians fitted to the reconstructed beads in the x, y, and z planes for both the 1-SOS and 2-SOS reconstructions.

Figure 6 shows the MIPs along the y axis for the 1-SOS and 2-SOS reconstructions. Both MIPs are normalized to their own maximum value. Figure 6 also presents the FWHM of the Gaussians fitted to the PSFs of the targets in x, y, and z as a function of depth. For both the 1-SOS and 2-SOS reconstruction, the deep-lying targets are reconstructed with higher amplitudes than the more superficially located targets. This is likely a result of the overlapping laser beams and reception fields of the detectors in the center of the imaging volume. The overlapping reception fields of the detectors can also explain why a decreasing FWHM with depth in the 2-SOS reconstruction is observed. For the 1-SOS reconstruction, an increasing trend in the FWHM was found since the reconstruction errors as a result of incorrect sound speeds accumulate with depth.

#### 3.2.3 Channel object

Two separate measurements on the channel object were performed to demonstrate its use in assessing the imaging depth. One measurement used 755 nm excitation and the other 1064 nm. During the measurements, the four channels of the object were filled India ink solutions with blood simulating optical absorption coefficients of 2.85 and $4.56\;{\mathrm{cm}}^{-1}$ for the 755- and 1064-nm measurement, respectively.^62^ Reconstructions were made with a 2-SOS model where a 1445 m/s sound speed was assigned to the test object volume and resulted in the best reconstructions assessed by eye. The SOS assigned to the water yielded 1497  m/s.

**Fig. 7:**
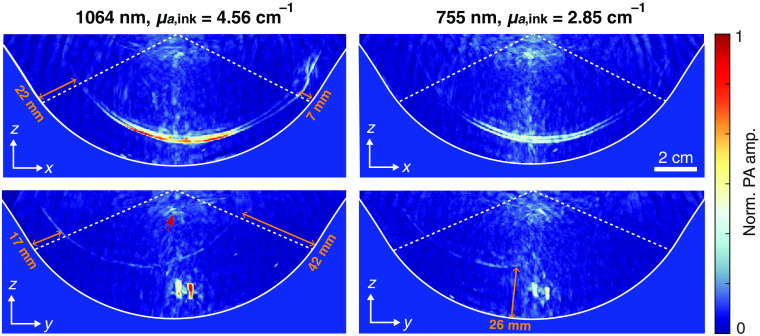
MIPs of the reconstructed channel phantom along the y and the x axes for measurements with two excitation wavelengths showing the two most superficially located channels. All reconstructions are normalized to the same value. The reconstructions are masked by the cup contour and the area in between the two dotted lines is the area where the channels gradually increase from the phantom’s surface. The distance of the channels to the object surface were measured and are shown in the reconstructions.

MIPs along the y and x axis of 2.4-cm thick subvolumes of the channel object centralized at y=0 and x=0, respectively, are presented in Fig. 7. All projections are normalized to the same value to compare the PA amplitude between the measurements. The most superficial channel was positioned in line with the xz plane and therefore shows in the MIP along the y axis, whereas the deeper-lying channel shows in the MIP along the x axis. Evident signatures from the two deepest channels were not observed in both reconstructions. The points deep inside the object (indicated by the red arrow in the 1064-nm measurement) presumably originate from parasitic PA signals.

The shortest distance to the surface was calculated for the channels at the start and end locations of the area where the distance to the surface gradually increases (area in between dashed lines in Fig. 7). The most superficial channel was found to start at 7 and ends at 22 mm and the second channel was found to start at 17 and ends at 42 mm. The most superficial channel is reconstructed as two curves, which is likely caused by a combination of limited optical penetration depths (±1  mm) in the ink solutions and by limited bandwidths of the detectors.

In line with the optical properties of the gel wax and the ink solutions, higher PA amplitudes are achieved at 1064 nm. The two largest channels can be observed entirely by eye for this wavelength, corresponding to an imaging depth of 42 mm. In the 755-nm measurement, the most superficial channel can also be observed entirely while the channel in the zy plane only shows up to a depth of 26 mm. The inhomogeneous illumination profile provided by the PAM 2 system on cup 8 can be observed in the reconstructions as higher PA amplitudes are observed around the rotational axis and at the upper sides of the channel. The amplitudes in the region in between those is lower, similarly as was seen on the reconstruction of black cup 8. This demonstrates that the imaging depth of multispectral systems and systems with an inhomogeneous illumination profile will be not only be wavelength-dependent but also spatially variant, which can make a generalized single value for the imaging depth unrepresentative for the entire imaging volume.

## 4 Discussion and Conclusion

The emergence of advanced 3D multispectral PA-US breast imaging systems calls for new systematic test materials and methods that can help in decision-making during system development, image quality assessment, regulatory evaluations, clinical translation, user training, and intersystem comparisons. In this work, we introduced a suite of test objects for standardized testing of multispectral tomographic PA-US systems equipped with a 3D detection aperture. In total, five test objects were designed of which two are for module specification verification studies. Three objects were developed to evaluate IQC being the PA spatial resolution and imaging depth, the spatial resolution and accuracy of the SOS maps obtained with US measurements, and the QPA ${\mathrm{SO}}_{2}$ accuracy.

The objects allow one to assess these characteristics in the imaging field of view as a function of settings in the measurement sequence or image reconstruction codes. Using a tomographic PA breast imaging system, the PAM 2 system, we demonstrated the PA appearance of the test objects and simultaneously demonstrated the value the test objects have in system design and imaging performance evaluation. Light distribution on the breast surface was visualized and showed to have the best homogeneity on the smaller cup sizes for the PAM 2 illumination geometry. The PA spatial resolution was shown to be depth-dependent, and we demonstrated the extent of the influence on reconstructed spatial resolution of errors in sound speeds used in the PA reconstruction. We also demonstrated that the PA imaging depth is wavelength-dependent and that assessing this characteristic in 3D systems is not obvious due to illumination geometries that often provide inhomogeneous fluence profiles on the object surface.

The applicability of the objects for assessing IQC related to SOS imaging and QPA could not be demonstrated in this paper since we did not have access to a system capable of acquiring and reconstructing such images. However, qualitative dual-wavelength imaging on the channel object carrying a blood-mimicking medium, showed wavelength-dependent amplitudes in the reconstructions, showing the necessity for QPA approaches, and demonstrating the utility of such a test object. The testing of fluence compensation algorithms or other QPA approaches to retrieve the ${\mathrm{SO}}_{2}$ can be done with multispectral imaging of such a test object carrying blood since the optical properties and geometries of all parts of the test object are known. The SOS object was imaged photoacoustically and was found to have a PA appearance as was expected (see Fig. S3 in the Supplementary Material) by only showing the PA targets embedded within it. This indicates that the acoustic interactions of the PA waves with the object are also as expected. Future work with a multispectral PA-US breast imager, however, has to demonstrate the true applicability of objects in SOS imaging and PA oximetry

Improvements can still be made in the designs of the objects to simplify the production protocols but also to improve the quality and applicability of the objects. The test objects as presented in this work contain PVCP, PAG, and gel wax. To simplify the reproduction of the objects, one of those soft tissue-mimicking materials could be selected to replace the other two. Second, the coatings manually applied to the subresolution beads can be irregular. As a result, not all beads generate similar PA pressures. Using commercially available subresolution targets with well-known diameters and made from a highly optically absorbing material is, therefore, recommended. This would also make the object suitable for testing the spatial variation in sensitivity accross the imaging FOV. To make the shapes of the wall-less channels in the SOS object more reproducible, 3D-printed channel shapes from water soluble plastics could be used instead of the modeling balloons. These 3D-printed placeholders can then be removed from the object by dissolving them in water after the PAG has solidified.

The optical and acoustic properties of the gel wax approximate the coefficients that can be expected in a real breast^63^^–^^70^ (see Fig. S4 in the Supplementary Material). The optical coefficients are a bit higher than what is found in the literature ($\pm 0.2\;{\mathrm{cm}}^{-1}$ for $\mu_{a}$ and $\pm 4\;{\mathrm{cm}}^{-1}$ for $\mu_{s}^{\prime }$) while the acoustic attenuation and sound speed are good correspondence. With this set of properties, the imaging depth was such that two of the four channels could be observed in the PA images. This makes the object suitable for system comparison studies to test and optimize QPA approaches and to search for the measurement settings resulting in an optimized imaging depth. In future work, more breast-like optical properties could be strived for, however, this object will never represent a real breast due to its simple architecture.

Although not described in this paper, one could think of more IQCs that can be quantified with this suite of test objects. Other characteristics can, however, require multiple measurements on one of the objects and may, therefore, be less convenient to quantify than the IQC covered in this paper. The sound speed contrast resolution and QPA contrast resolution are examples that are, respectively, the smallest sound speed or ${\mathrm{SO}}_{2}$ differences the system can measure. A possible approach for assessing the SOS contrast resolution is to fill the channels with liquids with different sound speeds just above the sound speed of the background, until these are just discernable in the sound speed images. A similar approach can be followed to assess the QPA contrast resolution, where channels are flushed with blood carrying different ${\mathrm{SO}}_{2}$ values, so as to find the smallest differences that the system can measure. We have to note that this requires the blood ${\mathrm{SO}}_{2}$ to be very well controlled during the measurements.

While designing the test objects, we built further upon existing designs and production protocols of test objects and phantoms reported by other groups. The detailed description and production protocols of our objects in this paper add new knowledge to this database and can be used as an inspiration for improved designs. In case the objects presented here are not suitable to test a system under development, adjustments can be made to the design like other object contours or PA target sizes. We believe that the test objects, as they are, also allow one to assess many of the performance measures of systems with other recording geometries. This, however, may need multiple measurements per object. For example in the case of linear arrays, imaging the PA point source will enable measuring the bandwidth of the detection elements and the PA spatial resolution of the system. The spatial variance in the resolution of the system can be assessed with a measurement on the PSF object when having several point targets in the field of view. The imaging depth can be assessed by making multiple acquisitions while moving the probe over the surface of the channel object to thereby acquire images with the channel located at different depths in the imaging plane. Similarly, the QPA accuracy, and the depth dependence of it, can be investigated when one or more channels are filled with blood or a blood mimicking liquid. Placing the probe on the surface of the SOS object allows one to test reflection mode SOS imaging.
