# Supplementary material for: Suite of 3D test objects for performance assessment of hybrid photoacoustic-ultrasound breast imaging systems
Source: J Biomed Opt. 2021 Dec 9;27(7):074709. doi: 10.1117/1.JBO.27.7.074709 (PMC8655513; doi:10.1117/1.JBO.27.7.074709)
Supplement: Supplementary file 1 [file JBO_027_074709_SD001.pdf]

Supplementary information

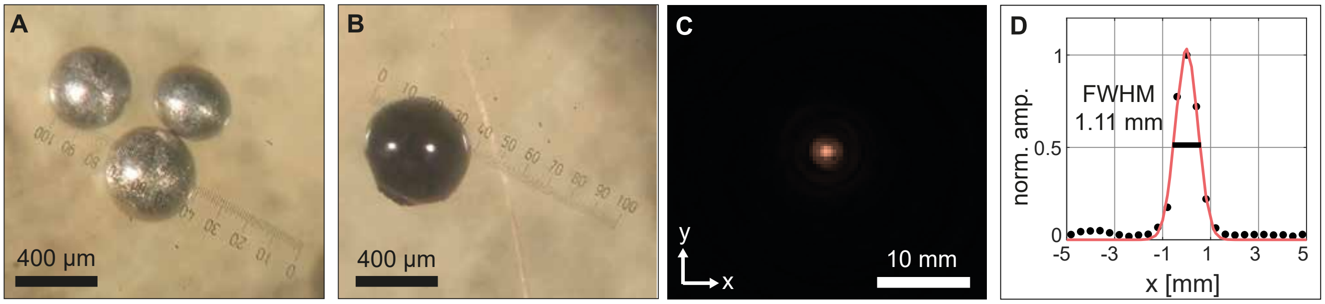

Figure S1. (A,B) Photographs of uncoated and coated beads, (C) MIP of measurement on a single bead with the PAM 2 System. For this measurement the bead was placed on an Agar slab and was not glued to the inner wall of the breast cup as in the procedure, (D) Gaussian fitted to the cross-section of the bead resulting in a FWHM of 1.1 mm.

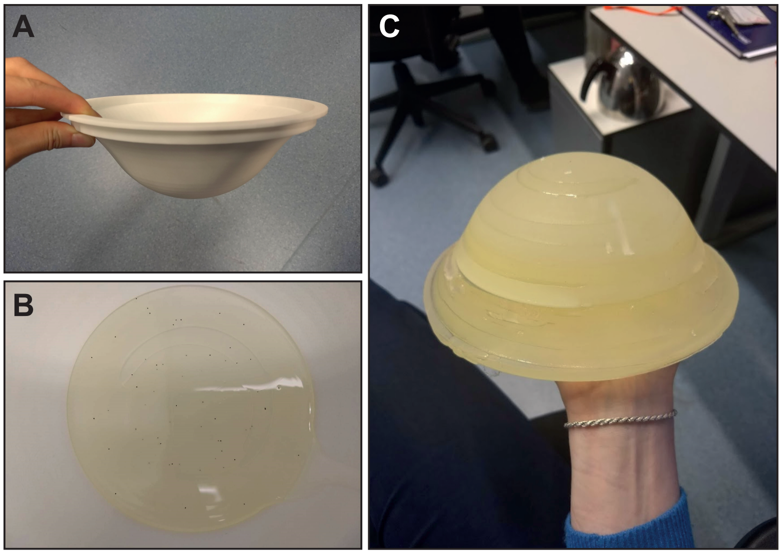

Figure S2. Pictures taken during the production of the PA spatial resolution object. (A) A picture of the nylon mould, (B) picture taken after distributing beads on top of one of the first PVCP layers, (C) the end result when removed from the mould, showing the different PVCP layers.

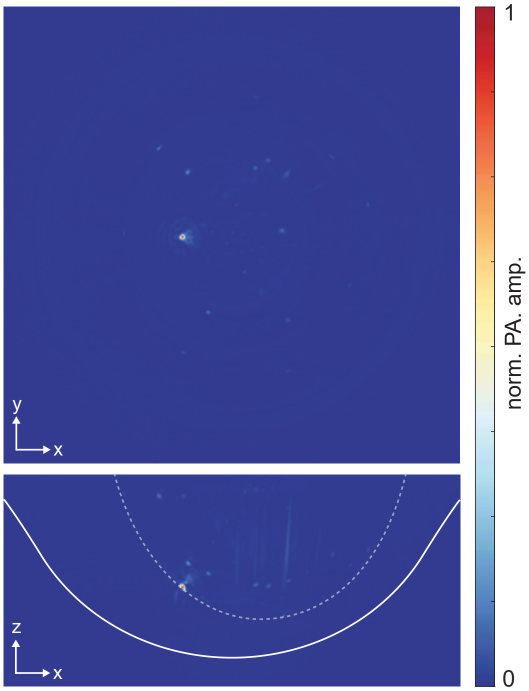

Figure S3. MIPs of the photoacoustic reconstruction of the SOS object showing the PA targets on the surface of the inner cup. The solid and dashed white lines are the contours of the outer and inner cups respectively. A 2-SOS model with 1496 m/s assigned to the water and 1485 m/s to the cup volume resulted in the best image.

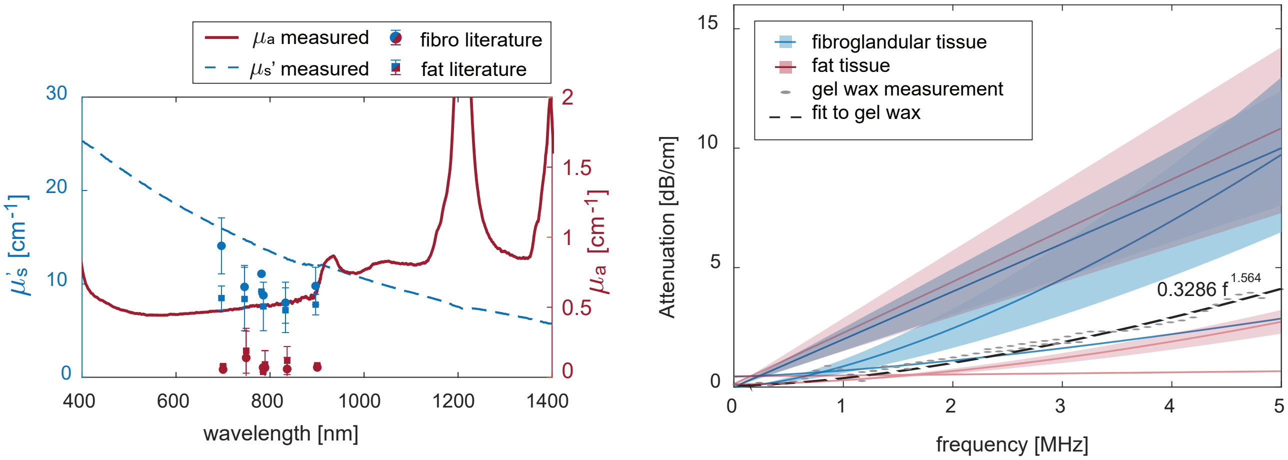

Figure S4. Reduced scattering and absorption coefficients and acoustic attenuation of the gelwax material used in the channel object. The powerlaw fit to the acoustic attenuation data is also plotted.
